# Di-*tert*-butyl­bis(*N*-isopropyl-*N*-methyl­dithio­carbamato-κ^2^ *S*,*S*′)tin(IV)

**DOI:** 10.1107/S1600536810007439

**Published:** 2010-03-03

**Authors:** Amirah Faizah Muthalib, Ibrahim Baba, Mohd Wahid Samsudin, Seik Weng Ng

**Affiliations:** a School of Chemical Sciences, Universiti Kebangbaan Malaysia, 43600 Bangi, Malaysia; b Department of Chemistry, University of Malaya, 50603 Kuala Lumpur, Malaysia

## Abstract

The dithio­carbamate anions in the title compound, [Sn(C_4_H_9_)_2_(C_5_H_10_NS_2_)_2_], chelate to the Sn^IV^ atom, which is six-coordinated in a skew-trapezoidal-bipyramidal geometry. The mol­ecule lies across a twofold rotation axis. The crystal studied was a non-merohedral twin, the ratio of the twin components being 0.82 (1):0.18 (1).

## Related literature

For the crystal structure of di(tert-but­yl)bis­(*N*,*N*-dimethyl­dithio­carbamato)tin(IV), see: Kim *et al.* (1987 ▶). For a discussion of the geometry of tin in diorganotin bis­chelates, see: Ng *et al.* (1987 ▶). For the treatment of non-merohedral twinning, see: Spek (2009 ▶).
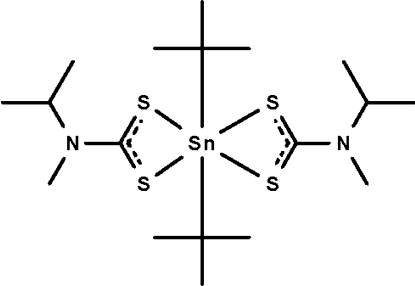


## Experimental

### 

#### Crystal data

[Sn(C_4_H_9_)_2_(C_5_H_10_NS_2_)_2_]
*M* *_r_* = 529.43
Monoclinic, 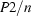

*a* = 11.2934 (11) Å
*b* = 7.0175 (7) Å
*c* = 15.6894 (15) Å
β = 95.016 (1)°
*V* = 1238.6 (2) Å^3^
*Z* = 2
Mo *K*α radiation
μ = 1.37 mm^−1^
*T* = 293 K
0.40 × 0.20 × 0.10 mm

#### Data collection

Bruker SMART APEX diffractometer
Absorption correction: multi-scan (*SADABS*; Sheldrick, 1996 ▶) *T*_min_ = 0.610, *T*_max_ = 0.875
7346 measured reflections
2838 independent reflections
2199 reflections with *I* > 2σ(*I*)
*R*_int_ = 0.065

#### Refinement

*R*[*F* ^2^ > 2σ(*F* ^2^)] = 0.062
*wR*(*F* ^2^) = 0.186
*S* = 1.09
2838 reflections
121 parameters
H-atom parameters constrained
Δρ_max_ = 1.76 e Å^−3^
Δρ_min_ = −1.58 e Å^−3^

### 

Data collection: *APEX2* (Bruker, 2009 ▶); cell refinement: *SAINT* (Bruker, 2009 ▶); data reduction: *SAINT*; program(s) used to solve structure: *SHELXS97* (Sheldrick, 2008 ▶); program(s) used to refine structure: *SHELXL97* (Sheldrick, 2008 ▶); molecular graphics: *X-SEED* (Barbour, 2001 ▶); software used to prepare material for publication: *publCIF* (Westrip, 2010 ▶).

## Supplementary Material

Crystal structure: contains datablocks global, I. DOI: 10.1107/S1600536810007439/ci5041sup1.cif

Structure factors: contains datablocks I. DOI: 10.1107/S1600536810007439/ci5041Isup2.hkl

Additional supplementary materials:  crystallographic information; 3D view; checkCIF report

## Figures and Tables

**Table:** 

Sn1—C1	2.233 (7)
Sn1—S1	2.5444 (18)
Sn1—S2	2.9911 (17)

**Table:** 

C1^i^—Sn1—C1	142.5 (4)

Symmetry code: (i) 
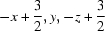
.

**Fig. 1.**
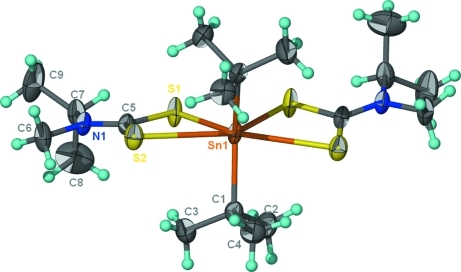
Displacement ellipsoid plot (Barbour, 2001) of [Sn(C6H5)2(C5H11NS2)2] at 50% probability level. H atoms are drawn as spheres of arbitrary radii. Unlabelled atoms are related to labelled atoms by the symmetry operation (3/2 - x, y, 3/2 - z).

## supplementary crystallographic information

### Experimental

Di-*t-*butyltin dichloride (10 mmol), isopropylmethylamine (10 mmol) and carbon disulfide (10 mmol) were reacted in ethanol (50 ml) at 277 K to produce a white solid. The mixture was stirred for 1 h. The solid was collected and recrystallized from ethanol.

### Refinement

H atoms were placed in calculated positions (C–H = 0.93–0.98 Å) and were included in the refinement in the riding model approximation, with *U_iso_*(H) set to 1.2-1.5*U_eq_*(C). The structure is a non-merohedral twin. The diffraction data were separated into two components by using *PLATON* (Spek, 2009). The final difference Fourier map had a peak near S2 and a hole near Sn1. The twin matrix is (0.293 0 0.707, 0 -1 0, 1.293 0 -0.293).

### Crystal data

**Table:**

[Sn(C_4_H_9_)_2_(C_5_H_10_NS_2_)_2_]	*F*(000) = 548
*M**_r_* = 529.43	*D*_x_ = 1.420 Mg m^−^^3^
Monoclinic, *P*2/*n*	Mo *K*α radiation, λ = 0.71073 Å
Hall symbol: -P 2yac	Cell parameters from 4020 reflections
*a* = 11.2934 (11) Å	θ = 2.6–28.1°
*b* = 7.0175 (7) Å	µ = 1.37 mm^−^^1^
*c* = 15.6894 (15) Å	*T* = 293 K
β = 95.016 (1)°	Block, colourless
*V* = 1238.6 (2) Å^3^	0.40 × 0.20 × 0.10 mm
*Z* = 2	

### Data collection

**Table:**

Bruker SMART APEX diffractometer	2838 independent reflections
Radiation source: fine-focus sealed tube	2199 reflections with *I* > 2σ(*I*)
graphite	*R*_int_ = 0.065
ω scans	θ_max_ = 27.5°, θ_min_ = 2.1°
Absorption correction: multi-scan (*SADABS*; Sheldrick, 1996)	*h* = −14→14
*T*_min_ = 0.610, *T*_max_ = 0.875	*k* = −8→9
7346 measured reflections	*l* = −11→20

### Refinement

**Table:**

Refinement on *F*^2^	Primary atom site location: structure-invariant direct methods
Least-squares matrix: full	Secondary atom site location: difference Fourier map
*R*[*F*^2^ > 2σ(*F*^2^)] = 0.062	Hydrogen site location: inferred from neighbouring sites
*wR*(*F*^2^) = 0.186	H-atom parameters constrained
*S* = 1.09	*w* = 1/[σ^2^(*F*_o_^2^) + (0.0968*P*)^2^ + 1.7978*P*] where *P* = (*F*_o_^2^ + 2*F*_c_^2^)/3
2838 reflections	(Δ/σ)_max_ = 0.001
121 parameters	Δρ_max_ = 1.76 e Å^−^^3^
0 restraints	Δρ_min_ = −1.58 e Å^−^^3^

### Fractional atomic coordinates and isotropic or equivalent isotropic displacement parameters (Å^2^)

**Table:**

	*x*	*y*	*z*	*U*_iso_*/*U*_eq_	
Sn1	0.7500	0.42189 (9)	0.7500	0.0369 (2)	
S1	0.77107 (19)	0.1454 (3)	0.64749 (11)	0.0514 (5)	
S2	0.82366 (17)	0.5223 (3)	0.57705 (11)	0.0469 (4)	
N1	0.8073 (5)	0.2052 (8)	0.4857 (3)	0.0402 (12)	
C1	0.5675 (6)	0.5241 (11)	0.7062 (4)	0.0426 (15)	
C2	0.4811 (7)	0.3967 (12)	0.7504 (5)	0.059 (2)	
H2A	0.4021	0.4474	0.7409	0.089*	
H2B	0.5043	0.3925	0.8107	0.089*	
H2C	0.4828	0.2702	0.7271	0.089*	
C3	0.5406 (8)	0.5079 (17)	0.6112 (5)	0.070 (2)	
H3A	0.4581	0.5351	0.5963	0.105*	
H3B	0.5580	0.3809	0.5932	0.105*	
H3C	0.5886	0.5972	0.5831	0.105*	
C4	0.5556 (7)	0.7310 (12)	0.7355 (6)	0.060 (2)	
H4A	0.4789	0.7793	0.7148	0.090*	
H4B	0.6164	0.8071	0.7131	0.090*	
H4C	0.5642	0.7362	0.7968	0.090*	
C5	0.8030 (5)	0.2889 (9)	0.5620 (4)	0.0373 (13)	
C6	0.8255 (8)	0.3238 (13)	0.4121 (5)	0.059 (2)	
H6A	0.7772	0.4361	0.4134	0.089*	
H6B	0.8038	0.2539	0.3605	0.089*	
H6C	0.9077	0.3598	0.4138	0.089*	
C7	0.7887 (7)	0.0018 (11)	0.4715 (5)	0.0501 (17)	
H7A	0.8045	−0.0620	0.5269	0.060*	
C8	0.6609 (11)	−0.0364 (16)	0.4406 (10)	0.102 (4)	
H8A	0.6102	0.0090	0.4822	0.153*	
H8B	0.6494	−0.1710	0.4325	0.153*	
H8C	0.6418	0.0283	0.3872	0.153*	
C9	0.8748 (12)	−0.0792 (14)	0.4117 (7)	0.097 (4)	
H9A	0.9505	−0.0174	0.4222	0.145*	
H9B	0.8442	−0.0579	0.3534	0.145*	
H9C	0.8841	−0.2136	0.4216	0.145*	

### Atomic displacement parameters (Å^2^)

**Table:**

	*U*^11^	*U*^22^	*U*^33^	*U*^12^	*U*^13^	*U*^23^
Sn1	0.0481 (4)	0.0382 (4)	0.0266 (3)	0.000	0.0163 (2)	0.000
S1	0.0883 (13)	0.0388 (9)	0.0311 (8)	−0.0034 (9)	0.0284 (8)	0.0010 (7)
S2	0.0678 (11)	0.0352 (9)	0.0400 (9)	−0.0041 (8)	0.0169 (8)	0.0007 (7)
N1	0.053 (3)	0.040 (3)	0.029 (3)	0.000 (2)	0.017 (2)	−0.001 (2)
C1	0.041 (3)	0.051 (4)	0.038 (3)	−0.004 (3)	0.010 (3)	0.004 (3)
C2	0.053 (4)	0.076 (6)	0.051 (4)	−0.014 (4)	0.015 (3)	0.001 (4)
C3	0.059 (5)	0.110 (8)	0.041 (4)	0.007 (5)	0.003 (4)	0.000 (5)
C4	0.064 (5)	0.052 (5)	0.064 (5)	0.009 (4)	0.005 (4)	0.004 (4)
C5	0.046 (3)	0.038 (3)	0.030 (3)	0.004 (3)	0.015 (2)	0.005 (3)
C6	0.087 (5)	0.060 (5)	0.033 (3)	−0.007 (4)	0.020 (4)	0.006 (3)
C7	0.080 (5)	0.037 (4)	0.035 (3)	−0.001 (4)	0.014 (3)	−0.003 (3)
C8	0.095 (8)	0.071 (7)	0.134 (12)	−0.026 (6)	−0.018 (8)	−0.007 (7)
C9	0.159 (12)	0.064 (7)	0.075 (7)	0.013 (6)	0.062 (7)	−0.014 (5)

### Geometric parameters (Å, °)

**Table:**

Sn1—C1^i^	2.233 (7)	C3—H3B	0.96
Sn1—C1	2.233 (7)	C3—H3C	0.96
Sn1—S1	2.5444 (18)	C4—H4A	0.96
Sn1—S1^i^	2.5444 (18)	C4—H4B	0.96
Sn1—S2^i^	2.9911 (17)	C4—H4C	0.96
Sn1—S2	2.9911 (17)	C6—H6A	0.96
S1—C5	1.739 (6)	C6—H6B	0.96
S2—C5	1.669 (7)	C6—H6C	0.96
N1—C5	1.338 (8)	C7—C8	1.506 (13)
N1—C6	1.453 (9)	C7—C9	1.520 (12)
N1—C7	1.457 (10)	C7—H7A	0.98
C1—C3	1.500 (10)	C8—H8A	0.96
C1—C2	1.533 (10)	C8—H8B	0.96
C1—C4	1.532 (11)	C8—H8C	0.96
C2—H2A	0.96	C9—H9A	0.96
C2—H2B	0.96	C9—H9B	0.96
C2—H2C	0.96	C9—H9C	0.96
C3—H3A	0.96		
			
C1^i^—Sn1—C1	142.5 (4)	H3A—C3—H3C	109.5
C1^i^—Sn1—S1	107.74 (18)	H3B—C3—H3C	109.5
C1—Sn1—S1	100.7 (2)	C1—C4—H4A	109.5
C1^i^—Sn1—S1^i^	100.7 (2)	C1—C4—H4B	109.5
C1—Sn1—S1^i^	107.74 (19)	H4A—C4—H4B	109.5
S1—Sn1—S1^i^	80.64 (8)	C1—C4—H4C	109.5
S1—Sn1—S2	63.73 (6)	H4A—C4—H4C	109.5
S1—Sn1—S2^i^	143.31 (6)	H4B—C4—H4C	109.5
C1—Sn1—S2	88.14 (17)	N1—C5—S2	122.8 (5)
C1^i^—Sn1—S2^i^	88.14 (17)	N1—C5—S1	117.5 (5)
S1^i^—Sn1—S2	143.31 (6)	S2—C5—S1	119.7 (4)
S2^i^—Sn1—S2	152.75 (6)	N1—C6—H6A	109.5
C1—Sn1—S2^i^	83.17 (17)	N1—C6—H6B	109.5
C1^i^—Sn1—S2	83.17 (17)	H6A—C6—H6B	109.5
S1^i^—Sn1—S2^i^	63.73 (6)	N1—C6—H6C	109.5
C5—S1—Sn1	94.8 (2)	H6A—C6—H6C	109.5
C5—N1—C6	118.6 (6)	H6B—C6—H6C	109.5
C5—N1—C7	123.4 (5)	N1—C7—C8	110.2 (7)
C6—N1—C7	117.9 (6)	N1—C7—C9	111.7 (7)
C3—C1—C2	108.9 (7)	C8—C7—C9	112.4 (9)
C3—C1—C4	110.8 (7)	N1—C7—H7A	107.4
C2—C1—C4	110.0 (6)	C8—C7—H7A	107.4
C3—C1—Sn1	112.5 (5)	C9—C7—H7A	107.4
C2—C1—Sn1	106.3 (5)	C7—C8—H8A	109.5
C4—C1—Sn1	108.3 (5)	C7—C8—H8B	109.5
C1—C2—H2A	109.5	H8A—C8—H8B	109.5
C1—C2—H2B	109.5	C7—C8—H8C	109.5
H2A—C2—H2B	109.5	H8A—C8—H8C	109.5
C1—C2—H2C	109.5	H8B—C8—H8C	109.5
H2A—C2—H2C	109.5	C7—C9—H9A	109.5
H2B—C2—H2C	109.5	C7—C9—H9B	109.5
C1—C3—H3A	109.5	H9A—C9—H9B	109.5
C1—C3—H3B	109.5	C7—C9—H9C	109.5
H3A—C3—H3B	109.5	H9A—C9—H9C	109.5
C1—C3—H3C	109.5	H9B—C9—H9C	109.5
			
C1^i^—Sn1—S1—C5	−76.5 (3)	S1^i^—Sn1—C1—C4	117.8 (5)
C1—Sn1—S1—C5	78.8 (3)	C6—N1—C5—S2	−2.8 (9)
S1^i^—Sn1—S1—C5	−174.8 (2)	C7—N1—C5—S2	−179.6 (5)
C1^i^—Sn1—C1—C3	103.1 (6)	C6—N1—C5—S1	175.8 (5)
S1—Sn1—C1—C3	−35.9 (6)	C7—N1—C5—S1	−1.0 (8)
S1^i^—Sn1—C1—C3	−119.4 (6)	Sn1—S1—C5—N1	−171.6 (5)
C1^i^—Sn1—C1—C2	−137.8 (5)	Sn1—S1—C5—S2	7.0 (4)
S1—Sn1—C1—C2	83.1 (5)	C5—N1—C7—C8	95.0 (9)
S1^i^—Sn1—C1—C2	−0.3 (5)	C6—N1—C7—C8	−81.9 (9)
C1^i^—Sn1—C1—C4	−19.7 (4)	C5—N1—C7—C9	−139.4 (8)
S1—Sn1—C1—C4	−158.7 (5)	C6—N1—C7—C9	43.8 (10)

Symmetry codes: (i) −*x*+3/2, *y*, −*z*+3/2.
